# Surgical abdomen masked by metabolic crisis: fulminant ischemic colitis in severe diabetic ketoacidosis - a case report and literature review

**DOI:** 10.3389/fendo.2026.1875153

**Published:** 2026-07-13

**Authors:** Jun Seong Chung, Jae Seung Kim, Jae Kyun Ju

**Affiliations:** 1 Department of Surgery, Chonnam National University Hospital, Gwangju, Republic of Korea; 2 Department of Surgery, Chonnam National University Hwasun Hospital, Hwasun, Republic of Korea; 3 Department of Surgery, Chonnam National University Medical School, Gwangju, Republic of Korea

**Keywords:** diabetic ketoacidosis, hyperosmolar hyperglycemic state, ischemic colitis, mesenteric ischemia, pneumatosis intestinalis, type 1 diabetes

## Abstract

**Background:**

Abdominal pain and gastrointestinal symptoms are common in diabetic ketoacidosis (DKA) and are generally reversible with metabolic correction. However, persistent or worsening abdominal findings should prompt concern for an underlying intra-abdominal emergency. Intestinal ischemia is an uncommon complication of severe hyperglycemic crisis in young patients but may progress rapidly to transmural necrosis due to profound hypovolemia and compromised microvascular perfusion.

**Case presentation:**

A 30-year-old woman with type 1 diabetes mellitus presented with a 3-day history of diarrhea, vomiting, and stupor following insulin omission. Laboratory evaluation demonstrated extreme hyperglycemia (1,869 mg/dL), severe metabolic acidosis (arterial pH 6.83; bicarbonate 2.2 mmol/L; anion gap 20.8 mmol/L), and acute kidney injury. She was managed for a mixed DKA and hyperosmolar hyperglycemic state with intravenous fluids, continuous insulin infusion, and electrolyte replacement. Although her anion gap narrowed, acidemia persisted, renal function deteriorated, and lactate levels increased. She subsequently developed hemodynamic instability requiring vasopressor support, along with progressive abdominal distension and rigidity. Non-contrast abdominal computed tomography revealed pneumatosis intestinalis extending from the ascending to the proximal transverse colon. She underwent emergency subtotal colectomy with end-loop ileostomy; histopathological examination confirmed transmural infarction of the ascending colon with subserosal abscess formation. Following intensive care management and continuous renal replacement therapy, she recovered and was discharged in stable condition.

**Conclusion:**

This case highlights a critical diagnostic challenge: apparent biochemical improvement during a hyperglycemic crisis, such as the narrowing of the anion gap, does not exclude evolving intestinal ischemia. Endocrinologists and critical care physicians must recognize that disproportionate or progressive abdominal signs require prompt imaging and early surgical consultation, even when contrast-enhanced studies are contraindicated.

## Introduction

Hyperglycemic crises remain a major cause of emergency admission, with mixed presentations of diabetic ketoacidosis (DKA) and hyperosmolar hyperglycemic state (HHS) frequently encountered in clinical practice. Abdominal pain is reported in up to 40-75% of patients with DKA and typically resolves following correction of metabolic derangements (1, 2). However, persistence or progressive abdominal pain necessitates prompt reassessment, as it may signify an underlying precipitating condition or a life-threatening complication rather than metabolic derangement alone (3). Intestinal ischemia, although uncommon, represents a life-threatening complication that can rapidly progress to transmural infarction, requiring early recognition and timely intervention (4, 5).

Here, we report a case of a young woman with type 1 diabetes who presented with a severe hyperglycemic crisis complicated by rapidly progressive acute ischemic colitis necessitating emergency subtotal colectomy. This case highlights a critical diagnostic challenge: apparent improvement in biochemical parameters, including narrowing of the anion gap, does not exclude evolving intestinal ischemia. We aim to highlight that worsening abdominal signs should prompt early surgical consultation and imaging in patients with worsening abdominal findings, despite apparent metabolic recovery.

## Case presentation

### Patient information

We present a case of a 30-year-old woman with type 1 diabetes mellitus, 8 months prior and receiving insulin therapy (insulin glargine 20U in the morning, insulin aspart 10U at each meal, for a total of 3 times) who presented to the emergency department with a 3 days history of diarrhea, recurrent vomiting, reduced oral intake, and progressive altered mental status. She reported preceding gastroenteritis-like symptoms and had withheld insulin during this period. Initial evaluation at an outside clinic revealed a capillary blood glucose level of 380 mg/dL, prompting transfer for a suspected hyperglycemic crisis.

### Clinical findings

On arrival, she was stuporous. Initial abdominal examination revealed a soft abdomen with mild tenderness in the right upper quadrant, but no rebound tenderness or rigidity. Vital signs were as follows: a blood pressure 110/60 mmHg, heart rate 130 beats/min, respiratory rate 20 breaths/min, temperature 37.5 °C, and oxygen saturation 97% on room air. Initial laboratory investigations demonstrated extreme hyperglycemia (1,869 mg/dL) and severe metabolic acidosis (an arterial pH of 6.83, bicarbonate of 2.2 mmol/L, and an anion gap of 20.8 mmol/L), consistent with severe DKA with suspected hyperosmolar features. Additional findings included acute kidney injury (AKI), with a blood urea nitrogen of 23.1 mg/dL, a creatinine 1.98 mg/dL, leukocytosis (20,500/µL), an elevated C-reactive protein (5.78 mg/dL), and a lactate level of 1.75 mmol/L (**Figure 1**).

**Figure 1:**
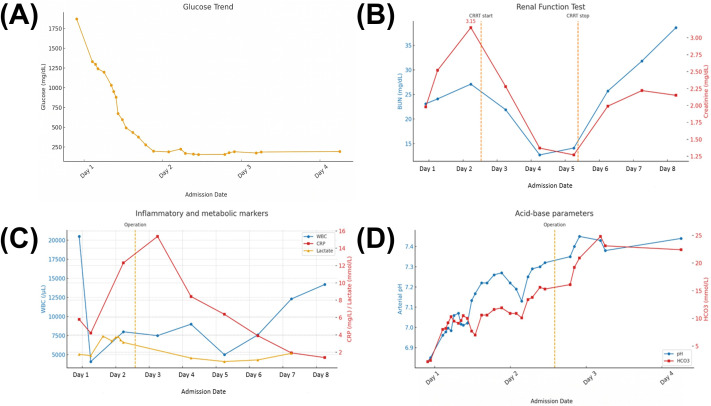
Serial trends in clinical and biochemical parameters during multidisciplinary management. **(A)** Temporal profile of blood glucose levels following hospital admission. **(B)** Changes in renal function (including blood urea nitrogen and creatinine) before and after initiation of continuous renal replacement therapy (CRRT). **(C)** Perioperative trends in inflammatory and metabolic markers (including white blood cell count, C-reactive protein, and lactate). **(D)** Perioperative changes in arterial pH and serum bicarbonate levels.

### Timeline

- Day 0 (Admission): Patient presented to the emergency department with stuporous mental status, tachycardia, mild fever, and severe hyperglycemia. Initial abdominal exam revealed a soft abdomen with mild right upper quadrant tenderness, without rebound tenderness or rigidity.
- Day 1 (Medical Management): Treated with intravenous fluid resuscitation, continuous insulin infusion, and electrolyte replacement, resulting in partial improvement of blood glucose levels and the anion gap.
- Day 2 (Clinical Deterioration): Patient condition worsened with persistent acidemia and severe abdominal pain. A non-contrast abdominal CT scan revealed pneumatosis intestinalis.
- Day 3 (Emergency Surgery): Emergency subtotal colectomy with an end-loop ileostomy was performed due to fulminant ischemic colitis.
- Day 4–9 (ICU Course): Admitted to the intensive care unit, requiring intubation and continuous renal replacement therapy (CRRT).
- Day 10–20 (Ward Course): Transferred to the general ward showing symptomatic improvement, followed by gradual feeding advancement.
- Day 21 (Discharge): Discharged home in stable condition.
- Follow-up Milestone: Successful ileostomy repair surgery was performed 16 weeks and 3 days after the initial procedure.

### Diagnostic assessment and therapeutic intervention

Following initial stabilization in the emergency department, she was admitted to the endocrinology service the following day and treated for combined DKA and HHS. Fluid resuscitation was aggressive initiated using 0.9% normal saline supplemented with potassium chloride, infused at a rate of 80 to 100 mL/hr via bilateral peripheral intravenous lines, alongside continuous insulin infusion and electrolyte replacement. The initial biochemical response indicated improving glycemic control, as blood glucose decreased gradually from 1,332 mg/dL to 198 mg/dL over the first day. Specifically, the patient’s anion gap, which was 20.8 mmol/L on hospital day 0, slightly decreased to 20.6 mmol/L, and subsequently normalized to 9.0 mmol/L on day 1. However, despite the definitive closure of the anion gap, severe metabolic acidosis persisted, with arterial pH near 7.0 and bicarbonate levels of 9–10 mmol/L. Concurrent with the large-volume resuscitation using 0.9% normal saline, her serum chloride levels steadily increased to 123 mEq/L on day 1 and 117 mEq/L on day 2, consistent with the development of a hyperchloremic non-anion gap metabolic acidosis.

By hospital day 2, her clinical condition deteriorated. Arterial blood gas analysis revealed persistent acidemia (pH 7.19 and bicarbonate 10.9 mmol/L) with rising lactate (3.8 mmol/L), and renal function worsened (creatinine 3.15 mg/dL). She developed an increasing oxygen requirement. Chest radiography demonstrated bilateral pleural effusions and pulmonary edema, for which an intravenous furosemide challenge (40 mg followed by 80 mg) was administered. Despite these interventions, she became hemodynamically unstable with persistent hypotension, an escalating norepinephrine requirement, and worsening acidosis, prompting initiation of an intravenous sodium bicarbonate infusion. Although the bicarbonate therapy produced transient improvement in her acid–base status and vasopressor requirements, her abdominal examination progressively worsened, revealing increasing distension, rigidity, and absent bowel sounds, alongside ongoing renal deterioration.

Given concern for an intra-abdominal catastrophe, a non-contrast abdominal computed tomography (CT) was performed due to renal impairment. Imaging revealed ischemic change with pneumatosis intestinalis involving the ascending to proximal transverse colon, with associated findings suggestive of peritonitis (**Figure 2**). Urgent surgical consultation was obtained, and in the setting of clinical deterioration and evolving peritoneal signs, emergency operative intervention was undertaken. Continuous renal replacement therapy (CRRT) was initiated preoperatively in view of progressive acute kidney injury and refractory metabolic acidosis.

**Figure 2:**
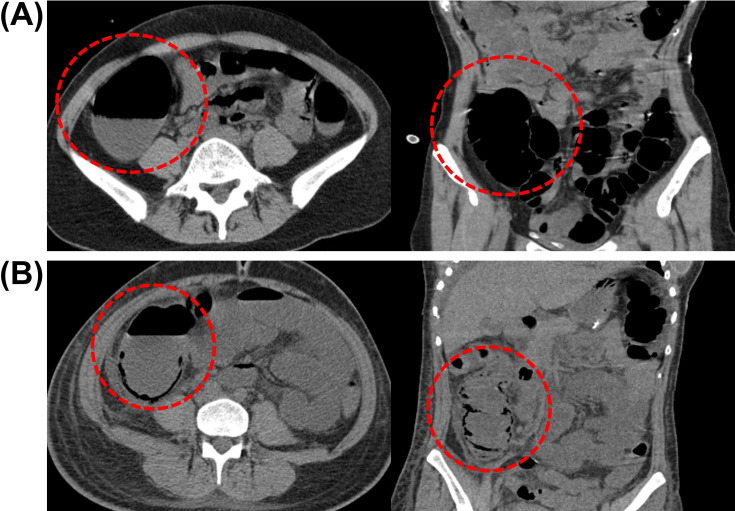
Radiological progression of acute ischemic colitis during a hyperglycemic crisis. **(A)** Initial abdominal computed tomography (CT) obtained at emergency department presentation demonstrated diffuse colonic dilatation. **(B)** Follow-up CT performed 48 hours after admission revealing newly developed pneumatosis intestinalis in the ascending colon, consistent with transmural infarction (red dotted circle).

She underwent emergency laparotomy, during which intraoperative findings revealed extensive ischemic changes that had dynamically progressed from the right colon and extended distally to the sigmoid colon. This generalized, multi-segment involvement necessitated a subtotal colectomy with end-loop ileostomy. Specifically, the operative field demonstrated diffuse serosal ischemia from the ascending to transverse colon, accompanied by abundant peritoneal fluid. Gross examination revealed necrosis involving the sigmoid colon and marked dilatation with black discoloration of the ascending colon (**Figure 3**). Histological examination revealed bowel ischemic change with serosal suppurative inflammation in the sigmoid colon and transmural infarction with degeneration and subserosal abscess formation in the ascending colon.

**Figure 3:**
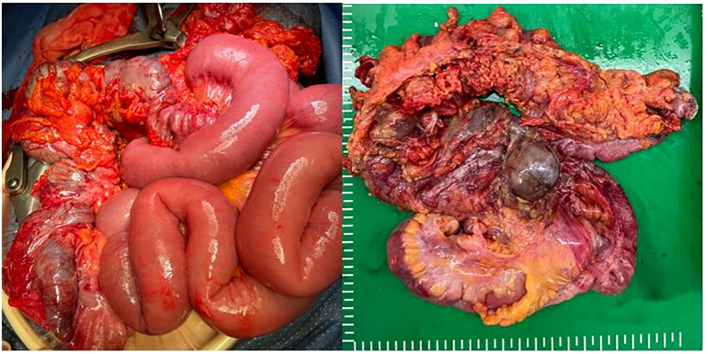
Gross pathological appearance of the subtotal colectomy specimen demonstrating extensive ischemic changes.

Post-operatively (3 days), she was managed in the intensive care unit with continued CRRT. Laboratory parameters subsequently reflected systemic improvement, with arterial pH 7.44, bicarbonate 26.2 mmol/L, lactate 0.92 mmol/L, and creatinine 1.27 mg/dL. The patient recovered and was discharged in stable condition on postoperative day 18. Furthermore, 16 weeks and 3 days after the initial surgery, an ileostomy repair was performed to restore intestinal continuity, which included terminal ileostomy resection and stapled anastomosis with the sigmoid colon using a gastrointestinal anastomosis stapling device. At long-term follow-up, she remained asymptomatic, achieved pregnancy, and underwent cesarean delivery with left cystectomy at 37 + 1 weeks’ gestation, 45 months after the initial surgery. She currently has no abdominal discomfort and is pregnant with her second child.

## Discussion

Abdominal pain is a common symptom of DKA; however, persistence or discordance with metabolic improvement should prompt immediate evaluation for severe intra-abdominal pathology. This case illustrates a key diagnostic challenge for clinicians during DKA resuscitation. Following treatment, hyperchloremic non-anion gap metabolic acidosis frequently develops as a result of aggressive crystalloid resuscitation and the preferential renal excretion of ketones (6). Specifically, the prolonged acidemia observed even after the closure of the anion gap was likely exacerbated by the large-volume resuscitation with 0.9% normal saline during the initial management of DKA. This well-recognized clinical consequence further complicated the interpretation of the patient’s metabolic recovery. This expected biochemical transition may obscure the clinical interpretation of ongoing acidemia, potentially delaying recognition of a concurrent ischemic process.

The pathophysiology of DKA-induced ischemic injury is multifactorial and overlaps with non-occlusive mesenteric ischemia (NOMI). Severe dehydration and osmotic diuresis result in marked hypovolemia, reducing mesenteric perfusion. Furthermore, the extreme DKA environment impairs microvascular autoregulation. Severe dehydration, increased blood viscosity, and systemic inflammation in DKA also promote a prothrombotic state, exacerbating microvascular compromise in the splanchnic bed (7). catecholamine surge exacerbates this process through intense splanchnic vasoconstriction. The right colon is particularly susceptible to such systemic hypoperfusion. According to the American College of Gastroenterology guidelines, an isolated right-sided colon is highly associated with systemic low-flow states and carries a poorer prognosis, often requiring immediate surgical intervention (4).

A limited number of published case reports have documented bowel ischemia occurring during, or despite apparent biochemical improvement of, hyperglycemic crises. As outlined in the Literature Comparison Table (**Table 1**), prior reports encompass small bowel ischemia with right colonic involvement (2), pediatric NOMI manifesting post-DKA resolution (8), toxic megacolon mimicking DKA in an adolescent (9), and recurrent NOMI associated with DKA in an adult with type 2 diabetes (10). Our case is distinct in that the ischemia, which initially manifested predominantly on the right side, dynamically progressed into a generalized, multi-segment NOMI in a young adult with type 1 diabetes, rapidly evolving to transmural necrosis without prior vascular disease.

**Table 1:** Literature comparison of diabetic ketoacidosis-related non-occlusive mesenteric ischemia.

Author year	Sex	Age (years)	Diabetes type	Site of ischemia	Mechanism/ key features	Management	Outcome
Kim HI et al. (2) (2025)	Male	54	T1DM	Small bowel + Rt. colon	Persistent pain during DKA; CT hypoenhancement	Surgery	Recovery
Frontino G et al. (8) (2022)	Female	13	T1DM	Small bowel (NOMI)	Pain persisted after correction; NOMI confirmed	Surgery	Recovery
Mansouri F (9) (2020)	Male	12	T1DM	Colon (cecum to sigmoid)	Mimicked DKA; gangrenous, dilated colon found intraoperatively	Subtotal colectomy	Recovery
Silva M et al. (10) (2023)	Male	58	T2DM	Extensive small + large bowel	Recurrent DKA to hypoperfusion to NOMI	Surgery	Mortality
Present case	Female	30	T1DM	Colon (ascending to transverse)	DKA with persistent pain; pneumatosis, peritonitis	Subtotal colectomy	Recovery

DKA, Diabetic ketoacidosis; T1DM, Type 1 diabetes mellitus; T2DM, Type 2 diabetes mellitus; NOMI, Non-Occlusive Mesenteric Ischemia.

This case highlights the importance of early imaging and multidisciplinary intervention. On CT, findings such as pneumatosis intestinalis and reduced mural enhancement during a hyperglycemic crisis should raise immediate concern for bowel necrosis. Current World Society of Emergency Surgery guidelines recommend urgent abdominal imaging in patients with persistent or disproportionate abdominal pain, irrespective of metabolic trends (11). As demonstrated in our proposed diagnostic algorithm (**Figure 4**), persistent pain despite initial DKA management should bypass conservative observation in favor of immediate imaging and surgical consultation. Overall, prompt recognition of the surgical abdomen masked by DKA and prompt subtotal colectomy were lifesaving in our patient.

**Figure 4:**
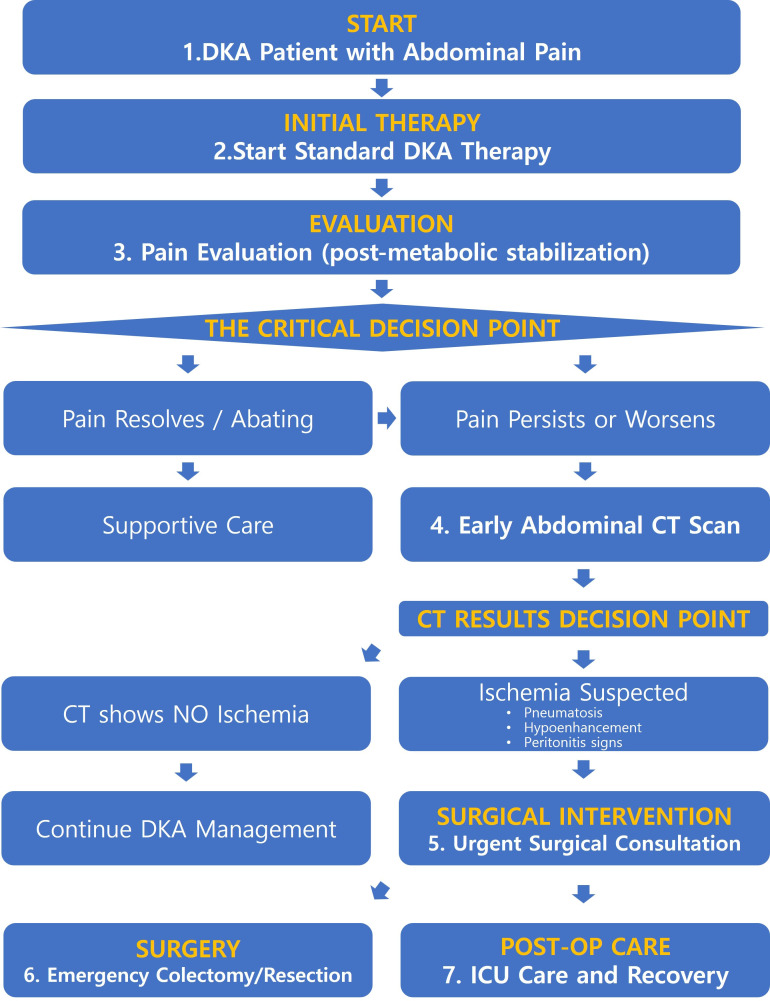
Proposed diagnostic algorithm for evaluation of abdominal pain in patients with diabetic ketoacidosis-associated.

Several limitations of this report should be acknowledged. First, as a single case report, it is inherently retrospective. Second, the patient’s initial AKI precluded the use of early contrast-enhanced CT. Consequently, it was difficult to accurately identify early radiologic hallmarks of colonic ischemia, such as mural hypoenhancement and pneumatosis intestinalis. Furthermore, because vascular patency could not be assessed, the presence of concurrent arterial or venous occlusive disease or thromboembolism cannot be definitively excluded. However, it must be emphasized that when there is a strong clinical suspicion of a life-threatening condition such as acute mesenteric ischemia, a contrast-enhanced CT should be urgently performed following a careful benefit-risk analysis; in such surgical emergencies, the critical survival benefit of an accurate diagnosis invariably outweighs the risk of contrast-associated AKI. Finally, the assessment of early circulating biomarkers specific to intestinal ischemia was limited during the initial presentation. Additionally, given the sheer severity of the presentation in a young patient, a comprehensive screening for underlying thrombophilia or a baseline hypercoagulable state was not performed postoperatively, which could have provided further context for her extensive ischemic events.

## Conclusion

Ischemic colitis is a rare but life-threatening vascular complication of severe DKA. This case highlights an important clinical principle: apparent metabolic improvement, including narrowing of the anion gap, does not exclude evolving intra-abdominal pathology. Clinicians must maintain a high index of suspicion for colonic ischemia when abdominal pain is disproportionate or persists despite adequate DKA resuscitation. Early multidisciplinary collaboration, prompt abdominal imaging, and surgical intervention are paramount to patient survival.

## Patient perspective

This patient had withheld her insulin injections for 3 days prior to presenting at our emergency department, owing to a decline in her general condition caused by diarrhea, vomiting, and poor oral intake. During this time, she did not monitor her blood glucose levels and hesitated to seek active attention for her enteritis. Having been diagnosed with type 1 diabetes only 8 months prior, and partly due to her young age, she did not fully appreciate the clinical importance of strict glycemic control. Following her successful recovery, she committed to strict adherence to her daily insulin regimen and frequent blood glucose monitoring, resolving to engage more proactively in her overall health management and clinical care.

## Data Availability

The original contributions presented in the study are included in the article/supplementary material. Further inquiries can be directed to the corresponding author.
